# The Mechanical Properties of Kevlar Fabric/Epoxy Composites Containing Aluminosilicates Modified with Quaternary Ammonium and Phosphonium Salts

**DOI:** 10.3390/ma13173726

**Published:** 2020-08-23

**Authors:** Rafał Oliwa

**Affiliations:** Faculty of Chemistry, Department of Polymer Composites, Rzeszow University of Technology, PL-35959 Rzeszow, Poland; oliwa@prz.edu.pl

**Keywords:** organoclay, epoxy composites, aramid fiber, mechanical properties, morphology, quaternary ammonium and phosphonium salts

## Abstract

We investigated the effect of modified aluminosilicates, including bentonite from Armenia (BA) modified with quaternary ammonium salts (BAQAS) and phosphonium salts (BAQPS), on the mechanical properties and morphology of Kevlar/epoxy composites. The Kevlar/epoxy composites containing 1.0 or 3.0 wt.% modified bentonites were fabricated using the hand lay-up technique. The mechanical properties, including the tensile, flexural, and in-plane shear strength, were tested. Based on the obtained results, we found that the mechanical properties increased with modified bentonite loading. The best results were obtained for composites containing 3 wt.% BAQAS, as most of the mechanical properties were significantly improved (tensile strength 302.9 MPa (+30%), Young’s modulus 16.3 GPa (+17%), flexural modulus 23.4 GPa (+12.5%), in-plane shear strength 22.8 MPa (+24.5%), and in-plane shear modulus 677.2 MPa (+42%)). The obtained improvements in the mechanical properties are attributed to the uniform dispersion of the filler, which was confirmed by the highest increase in the intergallery spacing, from 28.3 Å for BAQAS to 45.1 Å for the composite with 3 wt.% BAQAS. Scanning electron microscopy (SEM) analysis of the brittle fracture surface indicated that the addition of modified bentonite to the epoxy matrix changed the morphology of the Kevlar/epoxy/organoclay composites and improved the fiber–matrix interfacial adhesion.

## 1. Introduction

Among the composites with a polymer matrix, fiber reinforced composites have been widely used in the construction industry, and their applications are increasing. A continuous fiber polymer matrix composite offers high strength to weight, high toughness to weight, and design flexibility to match the material with the structural demands. The fibers are usually glass, carbon, or aramid, which do not break by brittle cracking. Kevlar fiber reinforced composites (KFCs) have been widely used as impact-resistant structures, such as in anti-ballistic applications, due to their high degree of toughness, associated with the failure mechanism, damage tolerance, and good impact performance [1,2,3,4]. Despite the excellent properties of KFCs, there are some disadvantages, such as low stiffness and compression strength, limiting their application in the aviation industry, shipbuilding, or sport goods. To increase the application capabilities of KFCs, many studies have investigated methods for improving the damage tolerance properties of aramid fiber/polymer composites. Extensive research has been devoted to the surface treatment of Kevlar fibers. Among the applied techniques, chemical grafting exhibits promising potential [5,6,7,8,9].

In order to obtain composites with better impact resistance, several researchers applied natural [10,11] and synthetic fibers [12,13] to prepared Kevlar hybrid composites. Yahaya and others [11] fabricated kenaf/Kevlar hybrid composites with different ratios of kenaf/Kevlar fibers. They showed that the maximum force to initiate penetration was higher in hybrid composites compared to kenaf/epoxy and Kevlar/epoxy composites. Hybridization of kenaf–Kevlar resulted in a positive effect in terms of the energy absorbed (penetration) and maximum load. Valença et al. [14] showed that glass/Kevlar hybrid composites were characterized by the highest mechanical properties, such as tensile and bending strength, tensile modulus, and impact strength. Zangana et al. have also applied, among others, glass and Kevlar fiber to create a hybrid trapezoidal corrugated composite core [15]. The result showed that the hybridization provides structures with better impact behaviour, without increasing structural weight.

Another way to enhance the interfacial interactions and mechanical properties of fiber reinforced composites is to improve the properties of the matrix by incorporating nanofillers into the polymer. Inorganic nanoparticles have gained use as potential reinforcing materials due to their low cost and ease of fabrication [16,17,18,19,20]. One of the methods of manufacturing flexible composite armor is the use of shear thickening fluid (STF) to impregnate Kevlar fibers [16,21]. Khodadadi et al. [22] used a mixture of polyethylene glycol (PEG) and silica nanoparticles to produce STF. They observed that the specific absorption energy (SEA) of Kevlar composites with 35 wt.% nanosilica was 2.3-times larger than those of neat fabrics. This source of improvement was traced to the formation of siloxane bonds between silica and PEG and the superior coating of Kevlar filaments with particles, which was confirmed by the pull-out test and SEM analysis.

Taraghi et al., [23] showed that the addition of 0.3 and 0.5 wt.% multi-walled carbon nanotubes (MWCNTs) to Kevlar/epoxy composites resulted in an increase in the absorbed energy capability by approximately 35% and 34% at ambient and low temperature, respectively. The Kevlar/epoxy composites with 0.5 wt.% MWCNTs exhibited improvements of 6%, 20%, 27%, and 48% in the tensile strength, Young’s modulus, flexural strength, and flexural modulus, respectively [24]. In turn, the authors of [25] modified the epoxy matrix in a seven-layer composite reinforced with weave aramid fabric using non-functionalized MWCNTs and -COOH functionalized MWCNTs. The described results showed that the addition of 0.32 wt.% MWCNTs with -COOH groups caused the highest increase of the thermal stability, tensile strength, and Young’s modulus of composites.

On the other hand, Reis and others [26,27,28] applied the cork powder or nanoclays Cloisite 30B to improve the impact resistance of Kevlar/epoxy composites. The filler content employed was 3 wt.% of the epoxy resin–hardener mixture. They observed that the maximum load increase was approximately 4.5% for laminates filled by cork, 10.4% for laminates filled by cork/clays, and 16.1% for laminates filled by clays. The elastic recuperation of nanoclay filled composites was approximately 40.1% higher than the control material. The cited publications indicated an increase in the energy absorption capacity of aramid composites containing nanoparticles including modified aluminosilicates.

Nanoclay has been explored worldwide as a cost-effective and potential filler for enhancing the mechanical properties of fiber reinforced polymer composites [29,30]. However, there is still a lack of information regarding the influence of nanoclay on the mechanical properties, such as the tensile strength, flexural strength, and shear strength, of aramid fiber reinforced composites. In our previous study, we observed an enhancement of the mechanical and thermal properties in epoxy composites [31] and unidirectional glass fabric reinforced epoxy composites [32]. The objective of the present paper was to use modified bentonites in aramid fibre reinforced composite technology, to extend their application as structural materials, by improving the stiffness and mechanical strength. For this purpose, 1.0 or 3.0 wt.% of bentonites modified with quaternary ammonium and phosphonium salts were added to the epoxy matrix. The influence of the type and content of modified bentonite on the mechanical properties and structure of obtained Kevlar fabric reinforced epoxy composites were investigated. The results of this publication will help to determine whether commonly known organoclays can be successfully used to modify Kevlar fibre-reinforced composites to obtain materials with higher mechanical parameters.

## 2. Experimental Part

### 2.1. Materials

The epoxy resin and the curing agent used in this work were Epidian**^®^** 624 (EP), containing mostly diglycidyl ether of bisphenol A and triethylenetetramine (Z1), respectively, both commercial grade products of Ciech-Sarzyna Plant, Nowa Sarzyna, Poland. Bentonite from Armenia (BA) was modified with phosphonium salts (BAQPS) using ethyltriphenylphosphonium bromide (Xiamen Pioneer Technology Inc., Xiamen, China) and modified with quaternary ammonium salts (BAQAS) using benzyl-C10-12-alkyldimethylammonium chloride (produced by Lonza, Basel, Switzerland). Kevlar fabric (plain, 173 g/m^2^) was purchased from Havel Composites, Svésedlice, Czech Republic.

### 2.2. Modification of Bentonite with Quaternary Ammonium and Phosphonium Salts

The procedure of modifying layered silicates with quaternary ammonium and phosphonium salts had been previously patented [33,34] and described in detail in a previous paper [32]. The procedure consists of introducing 30–40% aqueous solution of quaternary ammonium and phosphonium salts to a 10% suspension of bentonite in water heated to 60–90 °C, vigorous mixing for 1–3 h, removal of the supernatant liquid, and drying, grinding, and sieving to obtain modified bentonites of grain size below 63 µm.

### 2.3. Preparation of Epoxy Compositions

Epoxy compositions containing 1 and 3 wt.% modified bentonites were obtained. The modified bentonite was dispersed in the epoxy matrix using a four-step homogenization procedure [32], consisting of: premixing at room temperature by using a slow-running mechanical stirrer at velocity of 1500 rpm for 10 min, ultrasonic application at 50 °C for 10 min, mixing (at 50 °C, for 20 min) in a high-speed mixer (Dispermat CN40 produced by VMA-Getzmann, Gmbh, Reichshof, Germany) equipped with a turbine-like mixing blade, and final homogenization for 10 min in a high-speed shear rotating grinder at the rate of ca. 1000 s^−1^.

### 2.4. Preparation of Kevlar Fabric/Epoxy/Organoclay Composites

**Figure 1:**
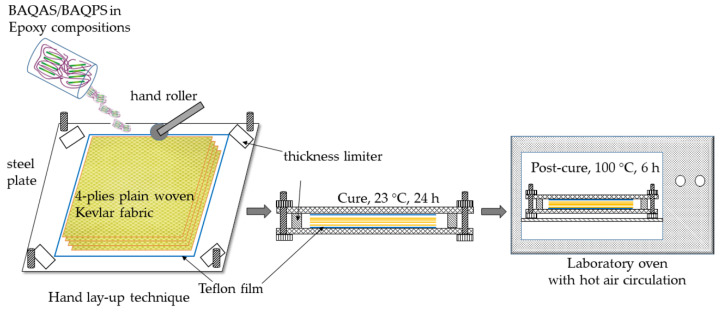
A schematic diagram of KFCs fabrication procedure.

The epoxy compositions containing modified smectic clay were used for the preparation of four-ply Kevlar/epoxy/organoclay composites using the hand lay-up technique (Figure 1). We added 12 wt.% hardener to the mixture, according to the resin manufacturer’s instructions. The epoxy compositions containing amine curing agent were poured on a Teflon film and one dry Kevlar fabric layer was impregnated using a hand roller. Then, another portion of the epoxy composition and dry fabric layer was stacked on it. The stacking procedures were repeated until the desired number of Kevlar fabric plies was laid. The uncured KFCs, after removing the excess of resin, was subsequently degassed for 5 min at room temperature in a laboratory vacuum chamber Vakuum UHG 400, (Schuechl, Bavaria, Germany). Then, the last layer was covered with another Teflon film before the sample was placed between two steel plates of dimensions 200 × 300 mm, which acted as a mold. To control the thickness of the KFCs, four steel plates with the required diameter were placed in the corners between the mold. The laminates (0F_4_) were left to cure at room temperature for 24 h and then post-cured in an oven with hot air circulation at 100 °C for 6 h. The resulting laminates contained ca. 38% of Kevlar fabric by weight. The samples were cut from the laminates with an oscillating cutting disk. The samples were used to measure the mechanical properties and microscopy analysis.

### 2.5. Morphology and Structure Analysis of Kevlar/Epoxy/Organoclay Composites

The brittle fracture morphology of the laminates was analyzed using scanning electron microscopy (Phenom ProX desktop SEM, Utrecht, The Netherlands). The fracture profiles were obtained after cooling in liquid nitrogen and an impact-break. The fractured profiles were copper sputter-coated before observation. The observations were conducted at 10 kV accelerating voltage of electrons with a 2500 and 7000× magnifications.

In order to determine the distance between the plates (d_001_) of modified bentonites and reinforced and non-reinforced epoxy composites with their addition, wide-angle X-ray scattering (WAXS) was used. The measurements were performed using a diffractometer Bruker Nanostar type (Bruker AXS, Inc. Madison, WI, USA) with a Cu lamp for the band width K_α_. The samples were in the form of plates 10 mm in width and 1.9 mm in thickness from the tested composites. The bentonite samples were tested in powder form.

The fiber surfaces from brittle fractures of composites were examined using an atomic force microscope (AFM) (Bruker Nano Surfaces Division, Santa Barbara, CA, USA) in Tapping mode. The influence of the modified bentonite for topography was investigated. The tests were performed using a Nanoscope V microscope (Bruker Nano Surfaces Division, Santa Barbara, CA, USA). The scanning speed was 1 kHz, and the resolution was 512 lines.

### 2.6. Study of Mechanical Properties

**Figure 2:**
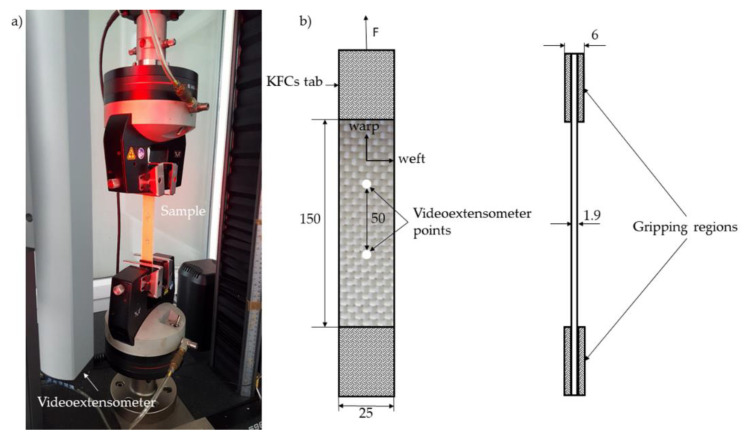
Tensile test setup (**a**) and schematic of tensile specimen geometry (in mm) with sample orientation (warp, weft) and applied force (F) (**b**).

Plate specimens (250 mm × 25 mm × 1.9 mm) were employed for the tension test. As shown in Figure 2, an Instron 5967 machine equipped with a videoextensometer was used to perform the tensile tests according to PN-EN ISO 527-4:2000 [35]. All specimens were tested at a speed of 2 mm/min.

**Figure 3:**
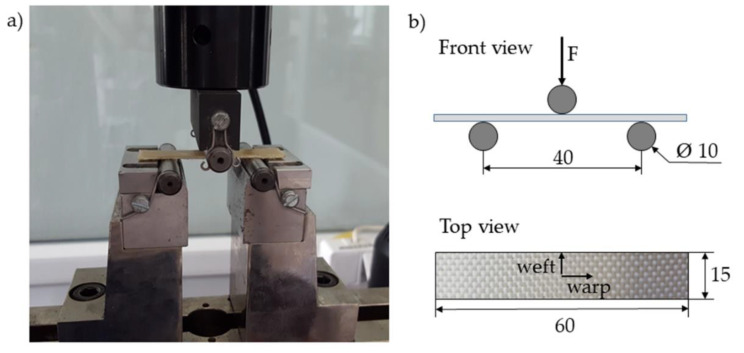
Flexural test setup (**a**) and schematic diagram of bending test fixture with specimen size (in mm), orientation (warp, weft) and applied force (F) (**b**).

The bending tests were performed according to PN-EN ISO 14125:2001 [36], using the same tensile machine equipped with a three-point bending rig. The vertical displacement speed of the rig was 1 mm/min during the test. The specimens were 60 mm long, 15 mm wide, and 1.9 mm thick, and the span was 40 mm, as shown in Figure 3.

**Figure 4:**
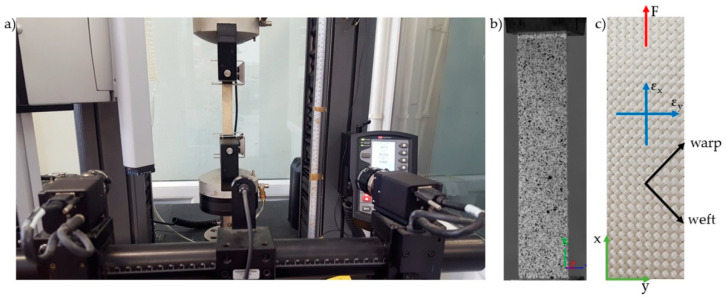
Experimental setup for in-plane shear test in a ±45° off-axis tension: view of the tensile machine with two cameras equipped Aramis system (**a**), in-plane shear sample covered with grey scale pattern (**b**), schematic representation of in-plane shear sample with sample orientation (warp, weft, x, y), applied force (F) and measured strains (ε_x_, ε_y_) (**c**).

The in-plane shear strength of the KFCs was also characterized from a tensile test in the ±45° direction of the fiber according to PN-EN ISO 14129:2000 [37]. The specimens (250 mm × 25 mm × 1.9 mm) were tensioned at a cross-head speed of 5 mm/min using the Instron 5967 machine equipped with a digital image correlation system (Aramis, GOM, Braunschweig, Germany) to determine the longitudinal and transverse deformation. Due to the limited number of recorded frames during the test, the recording with the camera was carried out in the shear deformation range 0–5%. The Aramis system was equipped with two cameras with 35 mm lenses. Before the measurement, a characteristic pattern was applied to the sample surfaces using graphite in the spray. This random grey scale pattern was recognizable by the program and divided into small rectangles called facets, which can overlap. Each facet has one unique structure and coordinates assigned to it, so that when the sample is loaded, they are recognized in the following pictures. In this study, 15 × 15 pixels facets and a 5-pixel step size were used. The calibration showed a pixel scale deviation equal to 0.027. The experimental setup for ±45° off-axis tension test with digital image correlation system and compact in-plane shear specimen are shown in the Figure 4.

The in-plane shear stress was calculated according to PN-EN ISO 14129:2000 [37] by the following Equation (1):

(1) $$\tau_{12}=\frac{F}{2\cdot a\cdot b}$$

where *F* is the shear force, N, *a* is the thickness, mm, and *b* is the width of the specimen, mm.

The shear modulus of the composites was determined from the shear strain curve for the shear strain range of 0.002–0.005, using the Equation (2) as per PN-EN ISO 14129:2000:

(2) $$G_{12}=\frac{\tau_{12}^{\prime \prime }-\tau_{12}^{\prime }}{\gamma_{12}^{\prime \prime }-\gamma_{12}^{\prime }}$$

where $\tau_{12}^{1}$ denotes shear stress at shear strain $\gamma_{12}^{1}=0.002\;\mathrm{and}\;\tau_{12}^{2}$ is the shear stress at shear strain $\gamma_{12}^{2}=0.005$.

The shear strain was calculated by the relationship (Equations (3) and (4))

(3) $$\gamma_{12}^{1}=\varepsilon_{x}^{1}-\varepsilon_{y}^{1}$$

(4) $$\gamma_{12}^{2}=\varepsilon_{x}^{2}-\varepsilon_{y}^{2}$$

where ***ε_x_*** and ***ε_y_*** are the longitudinal and transverse strain, respectively.

The longitudinal and transverse normal strains were determined from the digital images correlation recorded with high-speed cameras during the test (Section 3.1.3).

## 3. Results

### 3.1. Mechanical Properties of Kevlar/Epoxy/Organoclay Composites

#### 3.1.1. Tensile Strength

**Figure 5:**
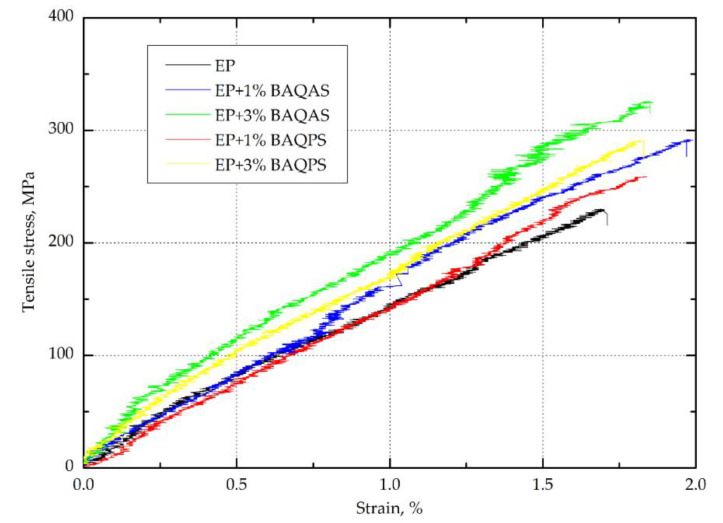
Representative stress-strain curves from the tensile test for the tested Kevlar-reinforced composites.

The results, i.e., the arithmetic means from ten tests of tensile strength for each aramid fabric--reinforced epoxy composites with modified bentonites are collected in Table 1. Figure 5 show a representative curve for each replicate test. The presented curves indicate non-linear tensile behavior of the composites with a clear point of specimen rupture after reaching the maximum stress. Furthermore, the elastic limit of the unmodified resin matrix composite is discrete and less visible than in other studies [11,38]. This may be due to the lower number of layers and the fibre content of the composite. A similar elastic deformation behaviour is presented by composites with 1 wt.% organoclays content, while composites with a matrix containing 3 wt.% of modified bentonites are characterized by a clear point of deviation from linearity. Moreover, curves indicate that composites containing modified bentonites characterized by higher deformation at break. Such tensile behavior of composites has affected the obtained results. It was found that the presence of modified aluminosilicates significantly improved the tensile strength of the Kevlar fabric reinforced hybrid composites. The highest, approximately 30%, increase of tensile stress was found for the laminates with matrices containing bentonites modified with quaternary ammonium salt. Surprisingly, the three times higher content of this organoclay in the epoxy matrix did not affects the change of this parameter. In comparison with the results of other authors concerning nanoclay [20,39] and other nanofillers [25], the obtained change was very pronounced, which may be related to the significant interlayer distance (d-spacing) of organoclay after modification and their proper dispersion in the epoxy matrix, particularly 3 wt.%, confirmed by the SEM analysis of the brittle fracture morphology of the composite.

In the case of composites with the addition of bentonites modified with quaternary phosphonium salt, the increase in the content resulted in an increase in the tensile stress, as a result of which, the composites with 1 and 3 wt.% of BAQPS were characterized by a higher tensile stress of approximately 11% and 22%, respectively, in comparison to the unmodified one. The obtained results were again consistent with the results of WAXS analysis. The composites containing BAQAS were characterized by higher values of the spacing between the organoclay platelets, which indicates that the epoxy chains have more easily penetrated between the silicate galleries, which results in an increase in the aspect ratio of bentonite layers. Better dispersion of this bentonite in the epoxy matrix leads to the absorption of polymer layers in the entire volume of the composite on the silicate particles [40,41]. As a result, the mobility of the polymer chains bound to the high aspect ratio platelets as well as those in intragallery is restricted during the load in the interfacial layer between the fibre and the matrix, leading to a better stress transfer to the fibres [42]. Therefore, a greater interlayer distance of BAQAS and a higher content of bentonite causes a local change in the polymer properties around individual bentonite platelets in a larger volume of the composite.

In turn, the Young’s modulus of composites containing 1 wt.% of modified bentonites was slightly decreased, compared to the reference sample, which may be associated with a slight dispersion, which may be related to the presence of areas with different clay levels (SEM analysis). While, the increase in content to 3 wt.% of BAQAS and BAQPS resulted in increases of the Young’s modulus values by approximately 17% and 10% respectively, compared to the EP/Kevlar sample. This enhancement could be attributed to the improvement of matrix stiffness [39], and change of properties by reducing residual stresses and improving the cohesive strength of the matrix [20], which is in accordance with our previous work related to organoclay/epoxy composites. This also confirms the stress–strain relationship curve in the elastic deformation range. The elastic limit of composites containing 3 wt.% is longer and is characterised by significantly higher stresses.

#### 3.1.2. Flexural Strength

**Figure 6:**
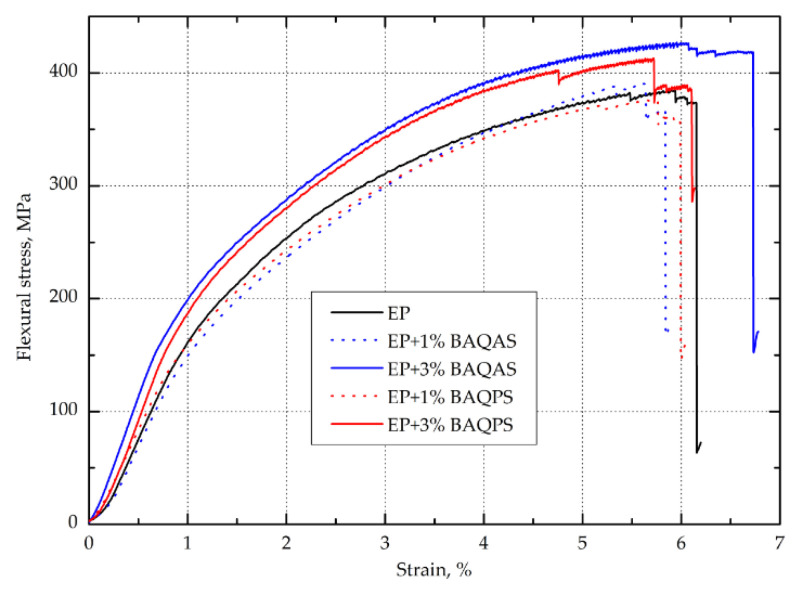
Representative stress-strain curves from the flexural test for the tested Kevlar-reinforced composites.

The results, i.e., arithmetic means from ten tests of flexural strength for each aramid fabric--reinforced epoxy composites with modified bentonites are collected in Table 2. Figure 6 show the tensile stress-strain relationship of Kevlar-reinforced composites. As in the case of the curves obtained during the tensile test, the curves presented in Figure 6 show differences in the performance compared to the relationships presented in other papers [11,38,43]. This time they concern the final part of the bending stress vs. deformation curves. Namely, our composites do not show high values of yield displacement and are characterized by failure in brittle manner, regardless of the type of matrix used. The results presented in Table 2. indicate that the addition of 1% wt. of modified bentonites to epoxy matrices did not significantly affect the bending stress and elastic modulus of laminates. This could be related to the fact that the longitudinal flexural strength is determined by the fiber properties. During the bending of continuous fiber reinforced composites, the composite is compressed and tensioned. Polymer composites, in particular aramid fiber composites, are characterized by the weak transverse properties [44,45] and significantly lower compression compared to tension strength [46]. As a result, the bending strength depends primarily on the compressive strength, as the damage usually starts on the compressive side [30]. In turn, scientific studies indicate that the addition of 1 wt.% of organoclay does not significantly affect the change in compression properties of epoxy nanocomposites [47,48,49,50]. As a result, the obtained composites with 1 wt.% of organoclay characterized by the bending properties at the level of unfilled composite. This is also visible in Figure 6, as the curves are similar to EP/Kevlar sample, with maximum stress, elastic and plastic limit and failure manner. In turn, the addition of 3 wt.% bentonite causes a slight change in failure manner, with highest yield displacement and maximum load. Furthermore, the slope of the curve is greater, which indicates greater rigidity. As a result of composites containing 3 wt.% modified BA, slight increases in the ultimate flexural strength and elastic modulus were observed.

The exception was the laminates with 3 wt.% BAQAS. The elastic modulus of this sample increased by 12.5% compared to reference sample, which coincides with the conclusions reached in the analysis of the Young’s modulus. Again, such reinforcing effects of the organoclay can be attributed to an appropriate dispersion and probably full intercalation of the high aspect ratio platelets which results in, that the interfacial interaction between aramid fibers and epoxy matrix can be improved. The obtained results showed that the aluminosilicates modified with quaternary ammonium and phosphonium salts did not affect the phenomenon of delamination, which is indicated as a major failure mechanism of continuous fiber reinforced composites, resulting in a deterioration in their strength [39]. This may be due to the appropriate modification and mixing process of the clay with the matrix to ensure a uniform dispersion. In addition, aramid composites tend to destroy the fiber surface skin [51,52], in contrast to carbon or glass reinforced composites where debonding of the fiber/matrix interface occurs [53,54]. This difference in aramid fibre structure described by Cheng and others in combination with the addition of organoclay may affect the phenomenon of delamination of obtained composites [55].

#### 3.1.3. In-Plane Shear Strength

**Figure 7:**
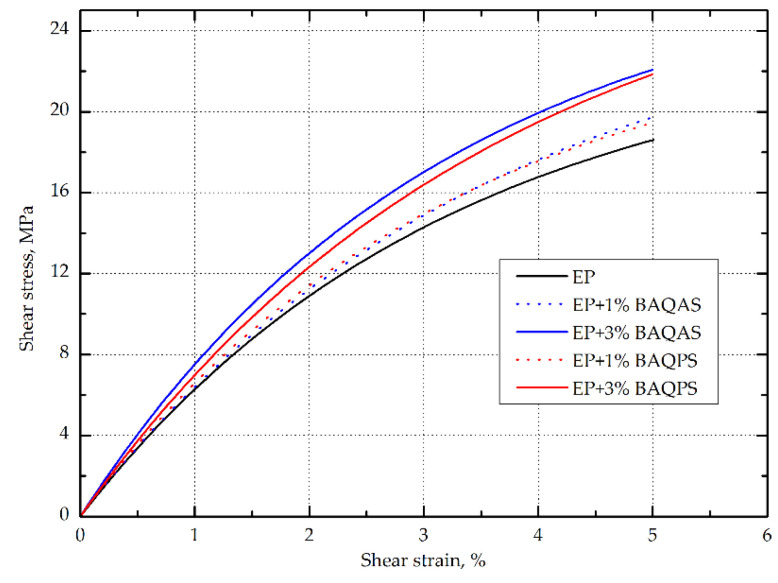
The shear stress vs. shear strain relationship in the range of 0–0.05 of Kevlar-reinforced laminates with EP and EP containing modified bentonite matrices.

The in-plane shear strength of hybrid aramid/epoxy composites was also investigated from tensile tests on (±45) laminate, which allows a uniform stress field over a large area of the sample [56]. The in-plane shear modulus in the range of shear strain of 0.002–0.005 and the shear strength at the point of shear strain of 0.05 according to the standard were also determined. The maximum shear strength and maximum shear displacement at the maximum shear load were determined. On the basis of the obtained results as summarized in Table 3, the addition of modified aluminosilicates significantly improved the behavior of the composite during tension at an angle of 45 degrees. In addition, an upward trend was observed as the clay content increased. As in the case of tensile and bending, higher values of the in-plane shear strength and in-plane shear modulus were obtained for the composites containing bentonite modified with quaternary ammonium salt. The composites containing 1 and 3 wt.% BAQPS showed 10.4% and 19.7% shear strength improvement, compared to the reference sample. In turn, for the composites containing 1 and 3 wt.% of BAQAS, an increase was recorded at the level of 12.6% and 24.6%, respectively, in relation to the composite with an unmodified epoxy resin (Figure 7). The obtained relationships confirmed that a larger d-spacing of BAQAS and appropriate dispersion provided better mechanical parameters.

**Figure 8:**
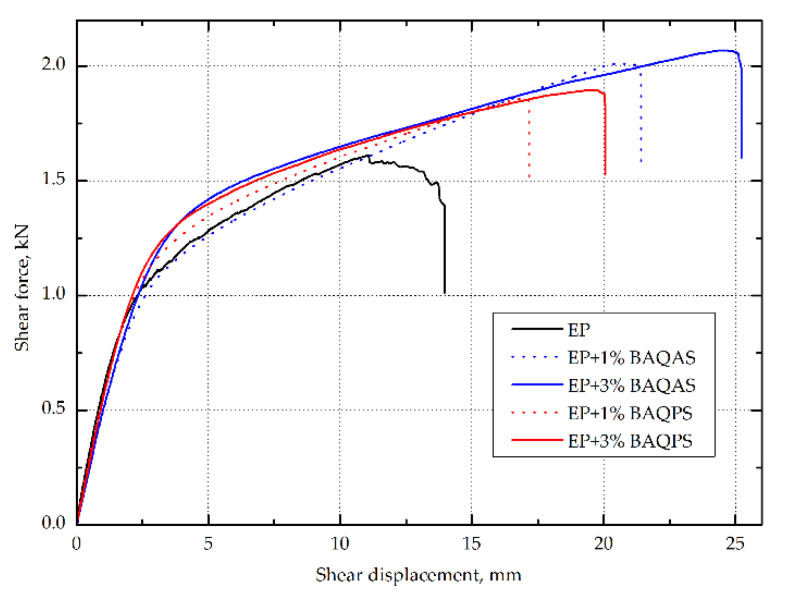
The shear load vs. shear displacement curves of Kevlar-reinforced laminates with EP and EP containing modified bentonite matrices.

The influence of the content of modified layered aluminosilicates in the epoxy matrix on the shear stress-shear displacement was even more visible after exceeding 0.05 for the shear strain value (shown in Figure 8). As a result, the maximum shear stresses of composites containing 1 and 3 wt.% BAQAS increased by 31.8% and 48.9%, respectively, compared to the unmodified composite. In turn, for laminates with 1 and 3 wt.% BAQPS content, improvements of the maximum shear strength by 20.6% and 26.2% were achieved compared to the reference sample, respectively. This improvement is attributed to the dispersion of clay in the matrix and the change in its properties, as the properties obtained under ±45 off-axis tension were dominated by the matrix properties and interface interaction [9,57]. On the recorded in-plane shear load–displacement curves of the composites, the slope of the curve decreased as the shear load increased, which indicates a reduction in the matrix stiffness due to local cracks (Figure 8) [58].

However, for composites containing modified bentonites, no plateau region of the shear curves and subsequent slow decrease of stress with an increase in the shear load, which is characteristic for aramid [9,59] as well as carbon [60] and glass [61] composites, was observed. In contrast, for composites with a modified matrix, the stress slightly increased as the deformation increased, until it ruptured. This effect was intensified by the fact that Kevlar fibers do not fail by brittle cracking, as opposed to glass and carbon fibers [26]. This was confirmed by the elongation values at the moment of specimen failure, which were significantly higher compared to the deformations obtained under on-axis tension. This non-linear behavior under the ±45 off-axis tension of composites reinforced with woven fabrics, which is related to the orientation of the fiber strands toward the loading direction, is very characteristic for these materials [61].

This indicates that, despite a significant increase in the stiffness of the matrix and the fact that there was no plateau, the addition of modified bentonites did not result in the rapid and brittle cracking of composites. Aramid composites containing modified bentonites were characterized by increased shear deformation at the break. The deformation of composites from BAQAS increased twofold, while that of BAQPS increased by half compared to the reference sample. This is worth noting, in particular since a significant change in the stiffness and behavior of the composites was observed even at lower strains, as shown by the values of the in-plane shear modulus. From the point of view of using aramid composites as energy-absorbing materials, for example, in car construction or in bulletproof vests, this is a beneficial effect [61,62,63].

**Figure 9:**
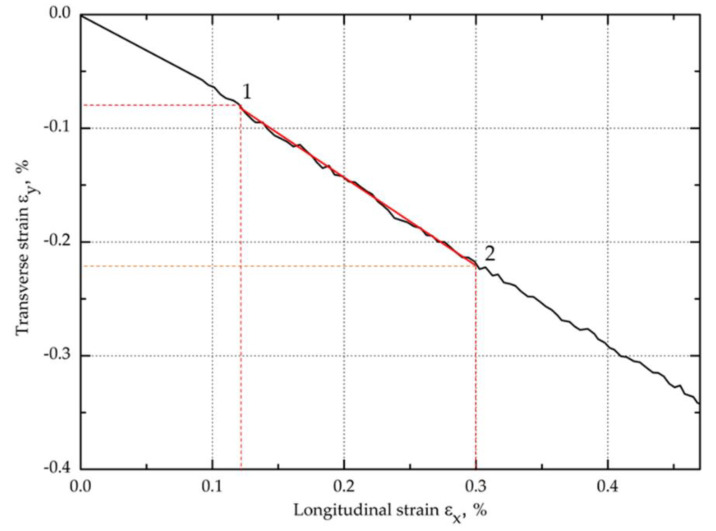
Example diagram of longitudinal strain vs. transverse strain obtained from digital image correlation.

**Figure 10:**
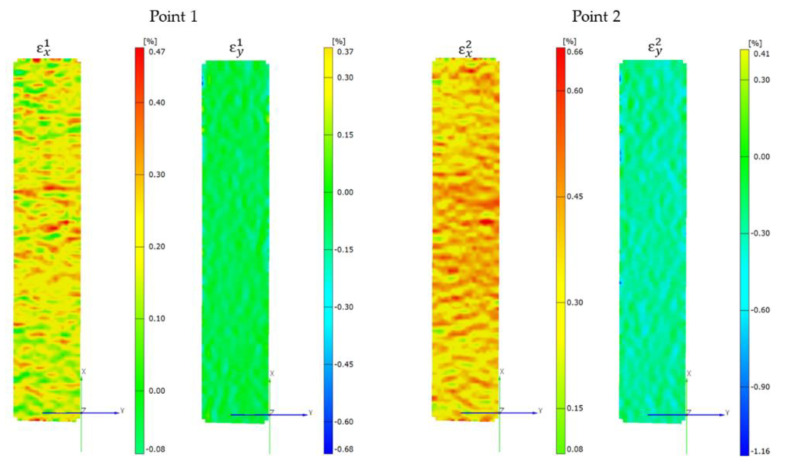
Representative longitudinal and transverse strains fields for two measurement points with a shear strain of 0.002 and 0.005, respectively.

**Figure 11:**
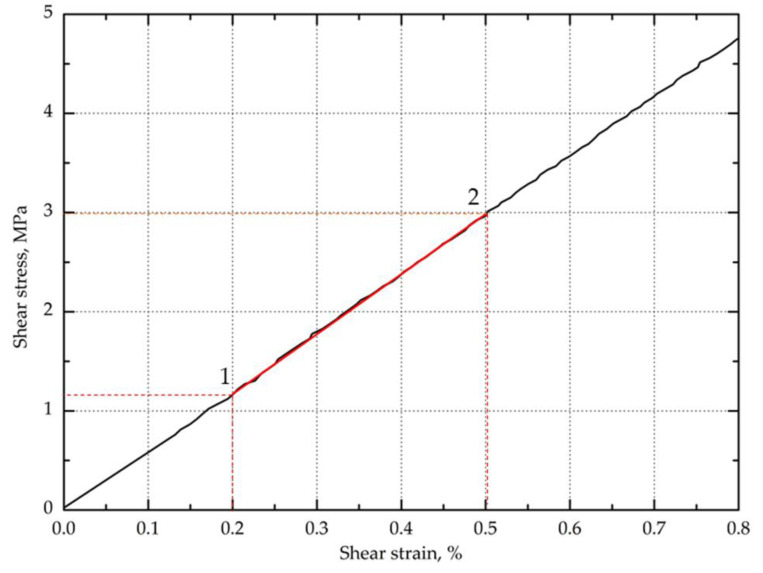
Representative shear stress-strain curve at the proportional limit.

The longitudinal ε_x_ and transverse ε_y_ normal strain values obtained from the analysis of images recorded during the study were used to calculate the shear strain (Figure 9). Examples of diagrams of longitudinal and transverse deformation fields for points 1 and 2 are shown in Figure 10. Then, on the basis of the data from the points 1 and 2 marked in Figure 11, the in-plane shear modulus from the shear stress–strain curve at the proportional limit was determined.

As demonstrated in Table 3, the shear modulus of the EP/Kevlar composite was 474 MPa, while, for composites containing 1% and 3% modified bentonites, the shear modulus values were approximately 25% and 42% higher, respectively, than those of the unfilled EP/Kevlar composite. The obtained results of the shear modulus confirm that the addition of aluminosilicates and their appropriate dispersion in matrices resulted in changes in the stiffness of the matrix and composites. As in the case of the Young’s and bending moduli, a more pronounced change was achieved with the addition of 3 wt.% bentonite.

### 3.2. Morphology and Structure of Kevlar/Epoxy/Organoclay Composites

#### 3.2.1. Analysis of Wide-Angle X-ray Diffraction

**Figure 12:**
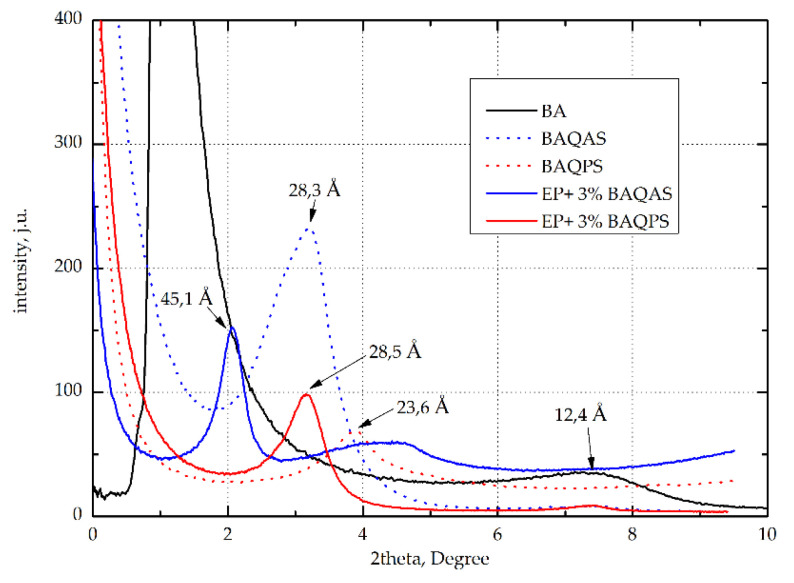
Wide-angle X-ray scattering (WAXS) curves of bentonites: unmodified BA, modified BAQAS and BAQPS, and cured epoxy compositions containing 3 wt.% BAQAS and BAQPS.

To assess the effectiveness of the modification of bentonites with QAS and QPS, the products were tested using X-ray scattering. Figure 12 shows the X-ray diffraction (XRD) patterns with the characteristic peaks of umodified clays, organoclays, and composites with 3 wt.% modified clays. As can be seen, the interlayer distance (d-spacing) of BA after modification increased by 11.6 Å and 8.2 Å, respectively. The larger distance between the layers of aluminosilicates modified with quaternary ammonium salt may be associated with the presence of the long alkyl chains. Phosphorus is characterized by lower electronegativity in relation to nitrogen, whereby the ion exchange capacity of quaternary phosphonium salts with intergallery ions is lower compared to quaternary ammonium salts.

This effect is also compounded due to a perceived shielding effect of the long alkyl chains, which affects the binding energy of the anionic components. As a consequence, quaternary ammonium salts penetrate better into galleries of aluminosilicates. This was confirmed by the curves recorded for composites EP containing 3 wt.% modified aluminosilicates, whose maximum peaks were shifted toward the lower diffraction angles. Among these composites, a much larger intergallery spacing, above 35 Å, was observed for the samples containing BAQAS. On the other hand, EP+3%BAQPS had a much smaller distance between the layers of 22 Å. Such a large difference may result from the presence of aromatic rings in the structure of phosphonic salt, which hinders the migration of epoxy resin chains to the inter-package spaces. Such a large difference is the effect of the smaller d-spacing of BAQPS plates in comparison to BAQAS, which makes the migration of polymer chains between clay packages more difficult.

**Figure 13:**
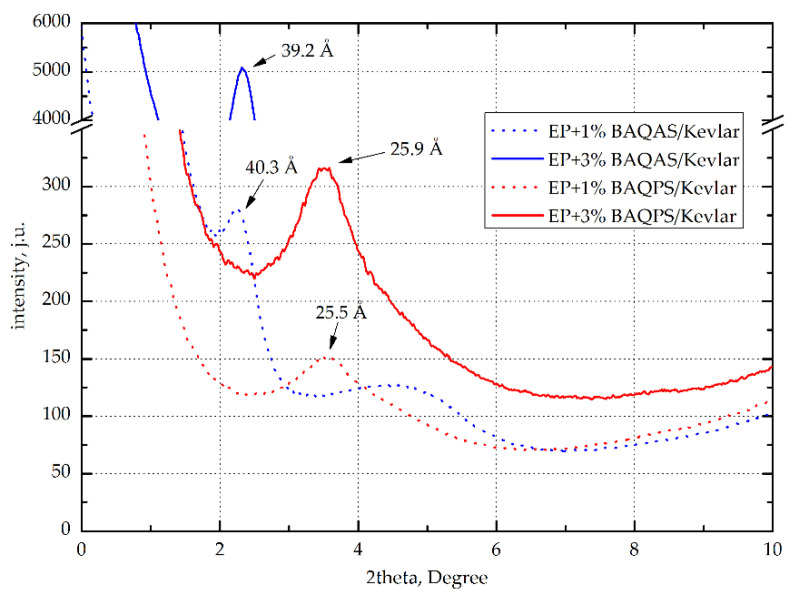
WAXS curves of epoxy/Kevlar composites with modified bentonites.

In the case of aramid fiber composites, the d-spacing of modified clays were lower than those for non-reinforced composites (Figure 13). This applies to materials with matrices containing 3 wt.% BAQAS and BAQPS, for which d_001_ were lower by approximately 5.9 Å and 2.6 Å, respectively. This may be due to the limited mobility of the clay platelets due to the presence of fabric. In addition, external pressure increased this effect, which contributed to the orientation of the clay platelets [64]. Differences may be due to the lower fiber content of the composite and the use of fabrics instead of mats. Lower fiber contents result in longer distances between the layers, which facilitates any arrangement of the clay platelets [65].

#### 3.2.2. SEM Analysis of Brittle Fracture Surface of Kevlar/Epoxy/Organoclays Composites

**Figure 14:**
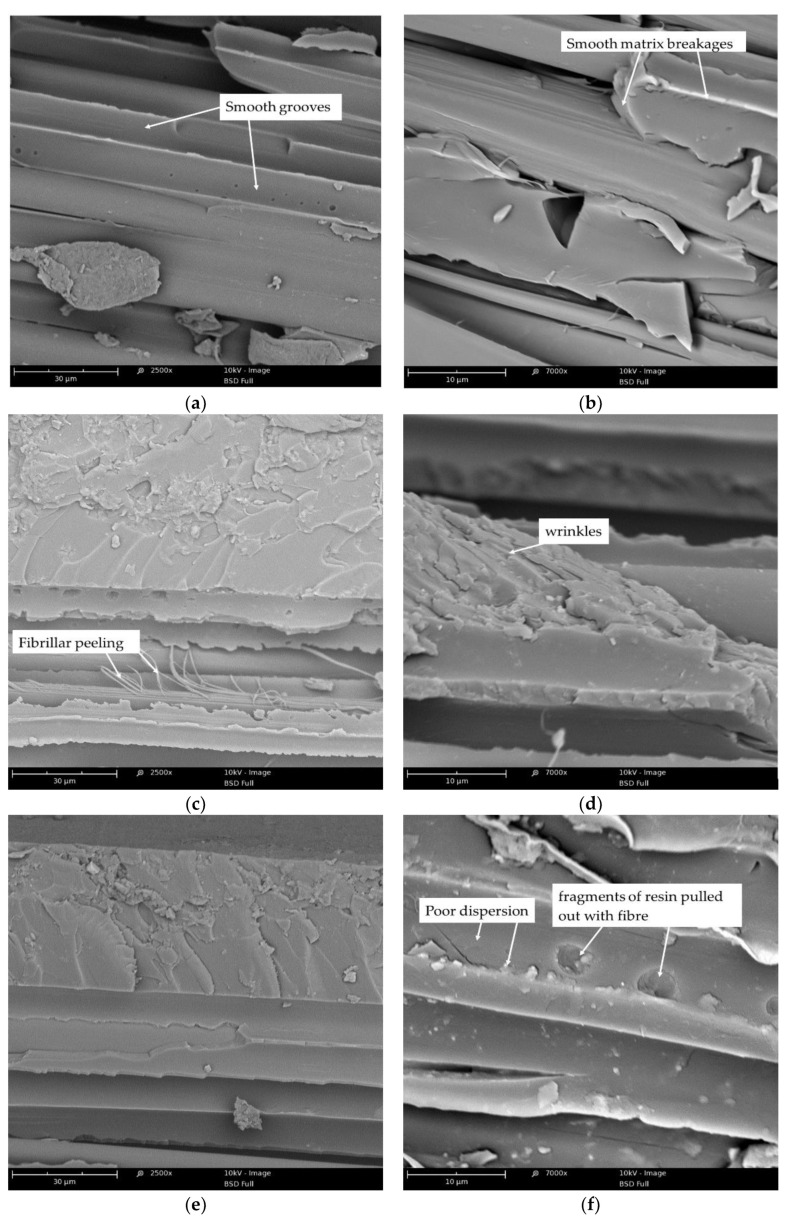

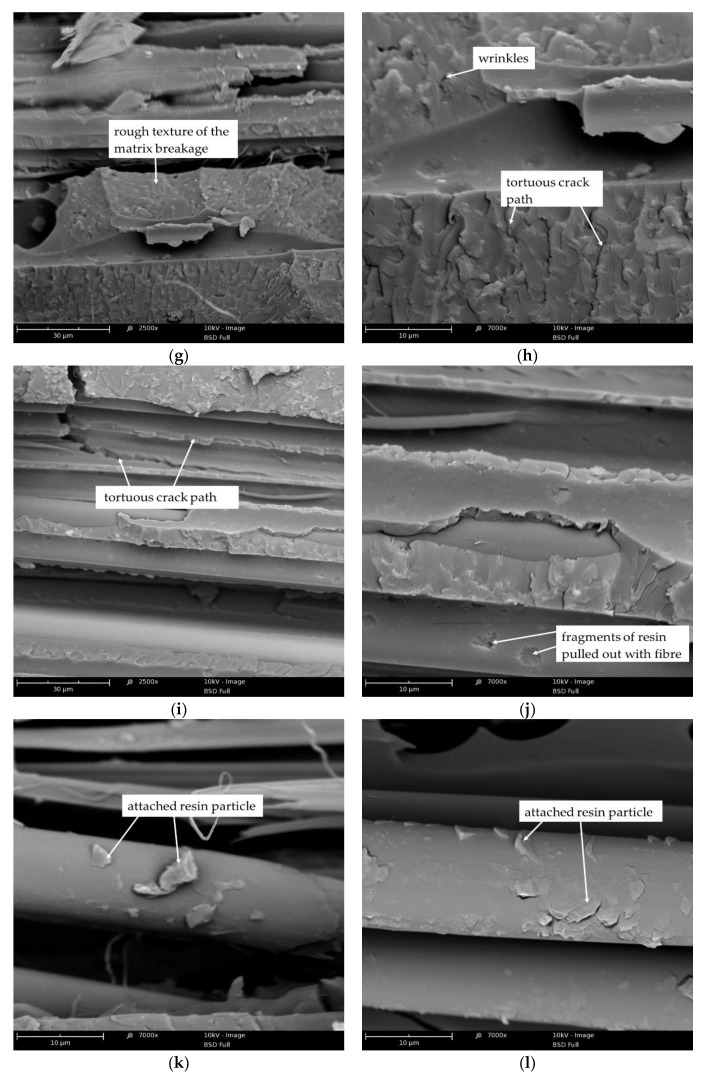
Scanning Electron Microscopy (SEM) microphotographs of brittle fractures of Kevlar-reinforced composites with matrices: EP (**a**,**b**), EP+1%BAQAS (**c**,**d**), EP+1%BAQPS (**e**,**f**), EP+3%BAQAS (**g**,**h**,**k**), EP+3%BAQPS (**i**,**j**,**l**).

Figure 14 show representative microphotographs of brittle fracture surfaces of Kevlar/epoxy composites and the Kevlar/epoxy/organoclay composites. Figure 14a,b shows topographies with features typical of a brittle mechanism of epoxy matrix/fiber composite cracking. Smooth surfaces are visible in the places where the fibers are pulled out, which indicates that there was easy interfacial debonding [66]. The interfacial debonding is likely promoted by the smooth cohesive matrix breakages. This suggests that the resistance for crack propagation is low, which results in weak mechanical properties. The SEM microphotographs of Kevlar/epoxy/organoclay composites exhibited changes in the morphology of these materials. In the case of composites containing 1 wt.% modified bentonite, a slight distribution of organoclay was observed, as evidenced by the increase in roughness and the created wrinkles (Figure 14c–e). In addition, fibrillas detached from the bulk of the fiber were identified (Figure 14c), which indicates that the failure mechanism of Kevlar/epoxy/organoclay composites was also related with so-called fibrillation [51,52].

This situation may occur when the shear strength between aramid chains is less or comparable to the shear strength between the matrix and fiber. Based on SEM images of composites containing 1% organoclay, we concluded that bentonite modified with quaternary ammonium salt exhibited better dispersion than when modified with phosphonium salt (Figure 14f). An observation at magnification indicated that BAQPS tended to agglomerate, which is likely due to the smaller d-spacing compared to BAQAS. On the other hand, increasing the amount of these bentonites to 3 wt.% caused a significant change in the structure and the formation of morphology with a structure characteristic for composites containing modified aluminosilicates (Figure 14g–j) [67,68].

The surfaces formed after brittle fracture of composites containing 3 wt.% modified aluminosilicates were more expanded and rougher. This extent of resin morphology may prevent crack propagation, by creating tortuous paths. As in the case of the reference sample, a debonding interface and cracking matrix in the SEM images of counterpart surfaces of Kevlar/EP+3%BAQAS and Kevlar/EP+3%BAQPS was identified. However, observation of the magnified images indicated that in fiber pull-out places, irregularly shaped craters were visible (Figure 14h,j). These small pieces of attached resin were visible at some points on the fiber surface, indicating good adhesion between the organoclay filled epoxy matrix and the fiber (Figure 14k,l). Improving the matrix–fiber interaction combined with the creation of tortuous crack propagation paths resulted in the improvement of the mechanical properties of these composites.

#### 3.2.3. Atomic Force Microscopy Analysis of Fiber Surface

**Figure 15:**
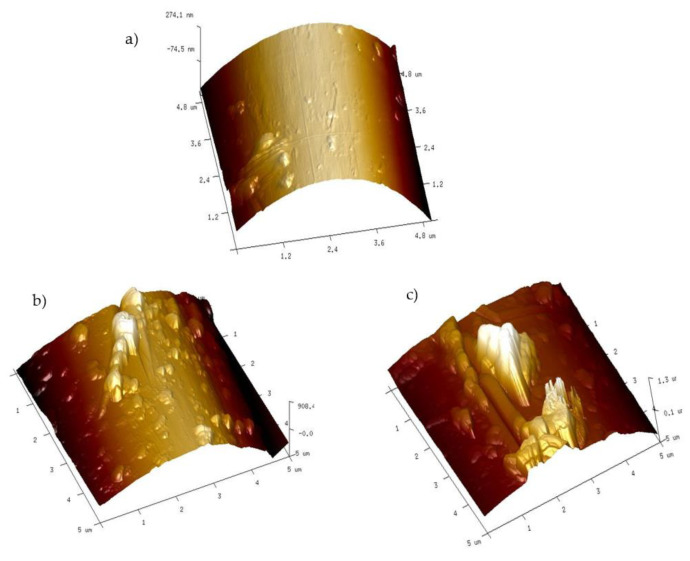
Atomic Force Microscopy (AFM) topography of a fiber pulled out after brittle fracture of the composites with matrices: EP (**a**), EP+3%BAQAS (**b**), EP+3%BAQPS (**c**).

A more detailed analysis of the topography of fibers pulled out after brittle fracture of composite samples performed with an AFM microscope confirmed the presence of resin fragments on the fiber surface. According to the literature data of AFM analysis, the surface of a Kevlar fiber is quite smooth with a small roughness to a maximum of several dozen nanometers [69,70]. In the case of fibers pulled from a composite with an unmodified resin matrix, small layers of resin are visible on its surface, with a maximum height and cross-section of 90 nm and 0.5 um, respectively (Figure 15a). On the other hand, the topography pictures of fibers from Kevlar/EP+3%BAQAS and Kevlar/EP+3%BAQPS composites show that the size of the adhered matrix pieces increased (Figure 15b,c). The analysis indicated that the heights were 0.5 µm and 1.2 µm and the cross-sections were 1 um and 1.5 µm, respectively. This confirms that the use of modified aluminosilicates changed the nature of the composite breakthrough as well as increased the interactions of the fibers with the polymer matrix, which resulted in the improvement of the mechanical parameters of aramid composites.

## 4. Conclusions

In this work, we obtained Kevlar-reinforced epoxy composites containing modified bentonites. The bentonites were modified with quaternary ammonium and phosphonium salts. The influence of the type and content of modified bentonites on the mechanical properties and structure of the obtained composites were investigated. On the basis of the obtained results, we found that both the amount and type of modified bentonites affected the mechanical properties of the composites. The mechanical properties improved with the loading of modified bentonites. On the other hand, the type of salts used to modify the bentonite had a more significant influence on the results obtained. The use of quaternary ammonium salt led to a distance between the plates of up to 28.3 Å, while bentonite modified with phosphonium salt had a d-spacing of 23 Å. This small difference affected the structure of the composites, as the addition of bentonites modified in this way to the epoxy matrix and their dispersion led to the spacing of BAQAS and BAQPS tiles at 40 Å and 25 Å, respectively. Such a large difference may result from the presence of aromatic rings in the structure of phosphonium salt, which hinders the migration of epoxy resin chains to the inter-package spaces. The larger inter-package spaces of BAQAS facilitates their dispersion in the polymer matrix, confirmed by SEM, which leads to an increase in the organoclay aspect ratio and direct absorption and binding to the polymer. As a result, the mobility of the polymer chains is limited, also in the interfacial layer fiber-polymer, which leads to a better stress transfer to the fibres in the composite. Higher clay content caused bigger changes in the whole volume of the composite. As a result, a significant improvement of mechanical properties of composites containing 3 wt.% of bentonite modified with ammonium salt was achieved. The obtained results of mechanical properties of composites reinforced with aramid fabric indicate that modified bentonites, which are commonly known as cheap and available polymer modifiers, are an important and interesting alternative in the context of improving the interaction between the polymer matrix and Kevlar fiber, which results in the improvement of functional properties of aramid-reinforced composites. Moreover, the addition of bentonites resulted in an improvement in the stiffness of composites and increase elongation at break, without causing a change in the manner of failure. Thus, aramid composites with epoxy resin matrix containing modified bentonites can be used not only as energy-absorbing materials but also as hybrid construction materials for example in aircraft body plating, airplane luggage compartments, ship hulls, kayaks etc.

## Figures and Tables

**Table 1:** The tensile properties of Kevlar reinforced composites ^1^. Epidian^®^ 624 (EP), bentonite from Armenia (BA) modified with phosphonium salts (BAQPS) and modified with quaternary ammonium salts (BAQAS).

Material	Ultimate Tensile Strength, MPa	Young’s Modulus, GPa	Elongation at Break, %
EP/Kevlar	233.9 ± 12.5	13.9 ± 1.2	1.6 ± 0.2
EP+1%BAQAS/Kevlar	303.1 ± 11.8	13.1 ± 0.9	1.9 ± 0.4
EP+3%BAQAS/Kevlar	302.9 ± 17.7	16.3 ± 3.0	1.6 ± 0.5
EP+1%BAQPS/Kevlar	260.3 ± 9.0	12.8 ± 1.0	1.8 ± 0.3
EP+3%BAQPS/Kevlar	285.7 ± 19.6	15.5 ± 0.2	1.7 ± 0.3

^1^ ± the standard deviation.

**Table 2:** The flexural properties of Kevlar reinforced composites ^1^.

Material	Ultimate Flexural Strength, MPa	Flexural Modulus, GPa	Elongation at Break, %
EP/Kevlar	389.9 ± 22.0	20.8 ± 0.8	6.3 ± 0.3
EP+1%BAQAS/Kevlar	381.0 ± 19.3	20.3 ± 0.6	6.1 ± 0.4
EP+3%BAQAS/Kevlar	401.5 ± 20.6	23.4 ± 0.9	6.6 ± 0.5
EP+1%BAQPS/Kevlar	379.8 ± 19.9	20.2 ± 1.0	6.0 ± 0.6
EP+3%BAQPS/Kevlar	394.9 ± 23.3	21.7 ± 0.9	6.6 ± 0.5

^1^ ± the standard deviation.

**Table 3:** Results of the in-plane shear test on (±45) laminates ^1^.

Material	Shear Strength at 5% Shear Strain, MPa	Shear Displacement at 5% Shear Strain, mm	Shear Modulus, MPa	Max Shear Strength, MPa	Max Shear Displacement, mm
EP/Kevlar	18.3 ± 1.0	3.53	474.7 ± 24.0	23.3 ± 1.1	12.5 ± 2.1
EP+1%BAQAS/Kevlar	20.6 ± 0.9	4.10	609.1 ± 26.6	30.7 ± 4.4	23.1 ± 2.4
EP+3%BAQAS/Kevlar	22.8 ± 0.8	4.18	674.0 ± 27.1	34.7 ± 3.1	25.9 ± 2.6
EP+1%BAQPS/Kevlar	20.2 ± 1.2	4.13	591.0 ± 19.6	28.1 ± 2.1	19.5 ± 2.3
EP+3%BAQPS/Kevlar	21.9 ± 0.6	4.23	677.0 ± 17.3	29.4 ± 3.3	17.4 ± 3.5

^1^ ± the standard deviation.
